# The Minimum Effective Analgesic Volume of 0.5% Bupivacaine for Ultrasound-Guided Anterior Suprascapular Nerve Block

**DOI:** 10.7759/cureus.31350

**Published:** 2022-11-10

**Authors:** Sami Kaan Coşarcan, Alper T Doğan, Özgür Koyuncu, Yavuz Gurkan, Ömür Erçelen

**Affiliations:** 1 Anesthesiology, Vehbi Koç Foundation (VKV) American Hospital, Istanbul, TUR; 2 Orthopaedics and Traumatology, Vehbi Koç Foundation (VKV) American Hospital, Istanbul, TUR; 3 Anesthesiology, Koç University, İstanbul, TUR

**Keywords:** ultrasound guided nerve block, diaphragm sparing nerve blocks, anterior suprascapular nerve block, postoperative pain, shoulder surgery, : regional anesthesia

## Abstract

Objective

The interscalene brachial plexus block (ISBB) constitutes the gold standard for analgesia after shoulder procedures. Ipsilateral phrenic nerve block remains the most common adverse effect after ISBB. Alternative nerve blocks are performed in shoulder surgery in order to prevent hemi-diaphragmatic paralysis (HDP). The purpose of the present study was to investigate the minimum effective local anesthetic volume of 0.5% bupivacaine for postoperative analgesia with an anterior suprascapular nerve block (ASSB). The secondary aim was to investigate diaphragm functions with the local anesthetic doses used while conducting effective volume research.

Method

This prospective observational study was conducted at the American Hospital of Istanbul, Turkey, from March to July 2022. The initial injected volume of 0.5% bupivacaine was 10 ml. Our clinical experience indicates that this yields a complete sensory block of the anterior suprascapular nerve. In accordance with the up-and-down method, the volume of 0.5% bupivacaine used for a particular patient was determined by the outcome of the preceding block, which represented block success. In case of effective ASSB being achieved, the volume of 0.5% bupivacaine to be administered to the next patient was lowered by 1 ml. In case of block failure, however, the volume of 0.5% bupivacaine to be applied in the subsequent case was increased by 1 ml. Ipsilateral hemi-diaphragmatic movement measurements were taken before (baseline) and 30 minutes after the block. General anesthesia was induced 60 minutes after the completion of the block performance by means of a standardized protocol.

Results

Sixty-seven patients were included in the study. The ED50 and ED95 calculated for anterior suprascapular nerve block using probit transformation and logistic regression analysis were 2.646 (95% CI, 0.877-2.890) and 3.043 ml (95% CI, 2.771-4.065), respectively. When complete paralysis was defined as 75% or above, partial paralysis as 25-50%, and no paralysis as 25% or less, volumes of 6 ml or lower appeared to cause no paralysis for the anterior suprascapular nerve block.

Conclusion

We, therefore, recommend using a volume of 6 ml or less in order to achieve diaphragm-sparing features for anterior suprascapular nerve blocks.

## Introduction

Shoulder surgery results in severe postoperative pain. Peripheral nerve blockade reduces pain scores and opioid consumption and increases patient satisfaction within the first 24 hours after shoulder surgery [1]. In the absence of peripheral nerve blocks, opioid consumption after shoulder surgery has been described as rivaling that recorded for thoracotomies [2,3]. Interscalene brachial plexus blocks (ISBB) constitute the gold standard for analgesia after shoulder procedures. ISBB results in lower pain scores and improves patient satisfaction compared with parenteral opioids [4]. ISBB is frequently employed in clinical practice since it provides anesthesia and analgesia to the shoulder as well as to the lateral aspects of the arm and the forearm. This results in lower opioid consumption and fewer subsequent opioid-associated adverse effects [5]. However, it is also reported to be associated with several complications and adverse sequelae, including phrenic nerve palsy (100%), recurrent laryngeal nerve block (3-21%), stellate ganglion block (5-75%; Horner’s syndrome), spinal (0.4-4%) and epidural anesthesia (2.2%), and convulsions (0.2-3%) when used at standard local anesthetic volumes of 20-30 ml. Phrenic nerve block has been linked to significant reductions in ventilatory function, such as a 21-34% decrease in forced vital capacity (FVC), a 17-37% decrease in forced expiratory volume, and a 15.4% decrease in peak expiratory flow rate [6]. The local anesthetic volume is therefore reduced in order to prevent ISBB complications. Ipsilateral phrenic nerve block remains the most common adverse effect after ISBB. Urmey et al. [7] demonstrated that ISBB results in a 100% incidence of hemi-diaphragmatic paralysis (HDP), as well as a 27% decrease in FVC and forced expiratory volume at one second. Although well tolerated by healthy subjects, HDP represents a prohibitive risk for patients with pulmonary pathologies, who may be unable to withstand the 30% reduction in FVC. Viable diaphragm-sparing alternatives to ISBB should achieve three separate and distinct goals: adequate surgical anesthesia (without general anesthesia), adequate postoperative analgesia, and a low incidence of HDP [8]. Alternative nerve blocks are performed in shoulder surgery in order to prevent HDP. Different approaches, such as suprascapular nerve blocks, costoclavicular blocks, shoulder blocks, supraclavicular blocks, and combined infraclavicular and suprascapular blocks, defined as diaphragm-sparing blocks, are therefore recommended [8,9].

The posterior approach is primarily described for suprascapular nerve blocks. This method is frequently employed for postoperative analgesia in shoulder surgery, especially after being described using the anterior approach. Various randomized, controlled studies have demonstrated postoperative analgesic efficacy. Diaphragm functions are also reported to be relatively well preserved compared to the interscalene block [10,11]. Different local anesthetic applications for the anterior suprascapular nerve have been described in the literature.

The primary outcome variable in this study was to determine the minimum effective anesthetic volume (MEAV50) of bupivacaine 0.5% in ASSB using the Dixon and Massey up-and-down method. The secondary outcome was to investigate diaphragm functions with the local anesthetic doses used while conducting effective volume research. We aimed to investigate local anesthetic doses capable of sparing diaphragm function while identifying the minimum effective dose of postoperative analgesia.

## Materials and methods

This interventional, double-blind trial was conducted in the American Hospital of Istanbul, Turkey, from March to July 2022. Written informed consent was provided by all participants.

Approval for the study was granted by the Koc University Clinical Research Ethics Committee (2022.003.IRB1.003) on February 2, 2022. The research was submitted to ClinicalTrials.gov (NCT05241977) on February 16. Patients started being included in the study on March 7. The actual primary completion date was June 3, and the study completion time was July 13.

Following receipt of ethics committee approval, adults (aged over 18) with American Society of Anesthesiologists Physical Status (ASA-PS) classifications I to III scheduled for unilateral arthroscopic shoulder surgery under general anesthesia were enrolled. All individuals provided written informed consent prior to taking part in this trial. In order to standardize the pain arising from surgery, only arthroscopic shoulder rotator cuff repair was included in the study.

Patients with bronchopulmonary disease, known phrenic nerve pathology, existing neurological deficits or neuropathy involving the surgical side of the brachial plexus, contraindications to nerve blocks (such as infection, bleeding diathesis, or allergy to local anesthetics), a history of significant psychiatric conditions capable of affecting the patient assessment, and pregnant women were excluded. Written informed consent was obtained from all patients. All blocks were performed by the same anesthesiologist (SKC) with high experience in regional anesthesia, and all diaphragm function measurements were taken by a different anesthesiologist (ATD) with high experience in regional anesthesia, blinded to the volume of the block.

The initial injected volume of 0.5% bupivacaine was 10 ml. Our clinical experience has shown that this volume of local anesthetic provides effective postoperative analgesia in an anterior suprascapular nerve block. Consistent with the up-and-down method by Dixon and Massey [12], the volume of 0.5% bupivacaine applied for a particular patient was based on the outcome of the preceding block, identified as representing block success as described down below. In case of effective ASSB being achieved, the volume of 0.5% bupivacaine planned for administration to the following patient was lowered by 1 ml. In contrast, in case of block failure, the volume of 0.5% bupivacaine in the subsequent case was instead increased by 1 ml.

An independent observer evaluated ipsilateral hemi-diaphragmatic movement by means of real-time M-mode ultrasonography using a 3.5-5 MHz curvilinear probe (GE, LOGIQ P9 R3, Seongnam-si, Republic of Korea). Patients were examined in the upright seated position. All participants were scanned using a low intercostal or subcostal approach, with either the liver or spleen being employed as an acoustic window. Diaphragmatic movement, commencing from the resting expiratory position as far as deep inspiration (sigh test), was recorded. The range of diaphragmatic movement was also recorded from the resting expiratory position during quick inspiration through the nose (sniff test). A decrease in hemi-diaphragmatic movement of more than 75%, no movement at all, or paradoxical movement were regarded as complete paresis. A decrease in hemi-diaphragmatic movement between 25% and 75% was regarded as partial paresis, and a decrease of less than 25% as no paresis. The extent of movement of the diaphragm was calculated in centimeters. Normal caudad movement was defined as positive (+) and paradoxical cephalad movement as negative (−). Each test was performed in triplicate, with the three values being averaged. All measurements were taken before (baseline) and 30 minutes after the block.

General anesthesia was induced 60 min after the completion of block performance using a standardized protocol consisting of intravenously administered propofol (2 mg kg^−1^), fentanyl (1 µg kg^−1^), and rocuronium (0.6 mg kg^−​​​​​​​1​​​​​​​^). Anesthesia was maintained with 1 MAC of desflurane and remifentanil infusion (0.05-0.20 µg/kg per min). Volume-controlled ventilation (6 ml/kg tidal volume) was applied through an endotracheal tube with a mixture of oxygen in the air (40/60%). Following surgery, rocuronium was antagonized with sugammadex 2 mg/kg. All patients received 1 g of intravenous (IV) paracetamol, 4 mg of dexamethasone, and 50 mg of dexketoprofen at the end of surgery.

Postoperative pain was assessed using a numerical rating scale (NRS) (from 0 [no pain] to 10 [worst possible pain]). Fentanyl was planned as rescue analgesia in the recovery room. In the case of NRS scores of 4 or more, patients were given 25 µg fentanyl IV, repeated if necessary (if NRS scores of 7 or more were present, patients were given 50 µg fentanyl IV). The total fentanyl dose administered was recorded in the recovery room. All patients were scheduled to receive intravenous morphine patient-controlled analgesia (PCA) (1 mg bolus dose; 10 minutes lockout only). Postoperative analgesia consisted of 1 g of oral paracetamol every 6 hours and 50 mg of dexketoprofen twice daily for 48 hours after surgery. All patients’ pain scores and total morphine consumption in the recovery unit (30 minutes and 1 hour) and ward follow-ups (6, 12, and 24 hours) in the postoperative period were recorded by the acute pain team.

Nerve block technique

**Figure 1:**
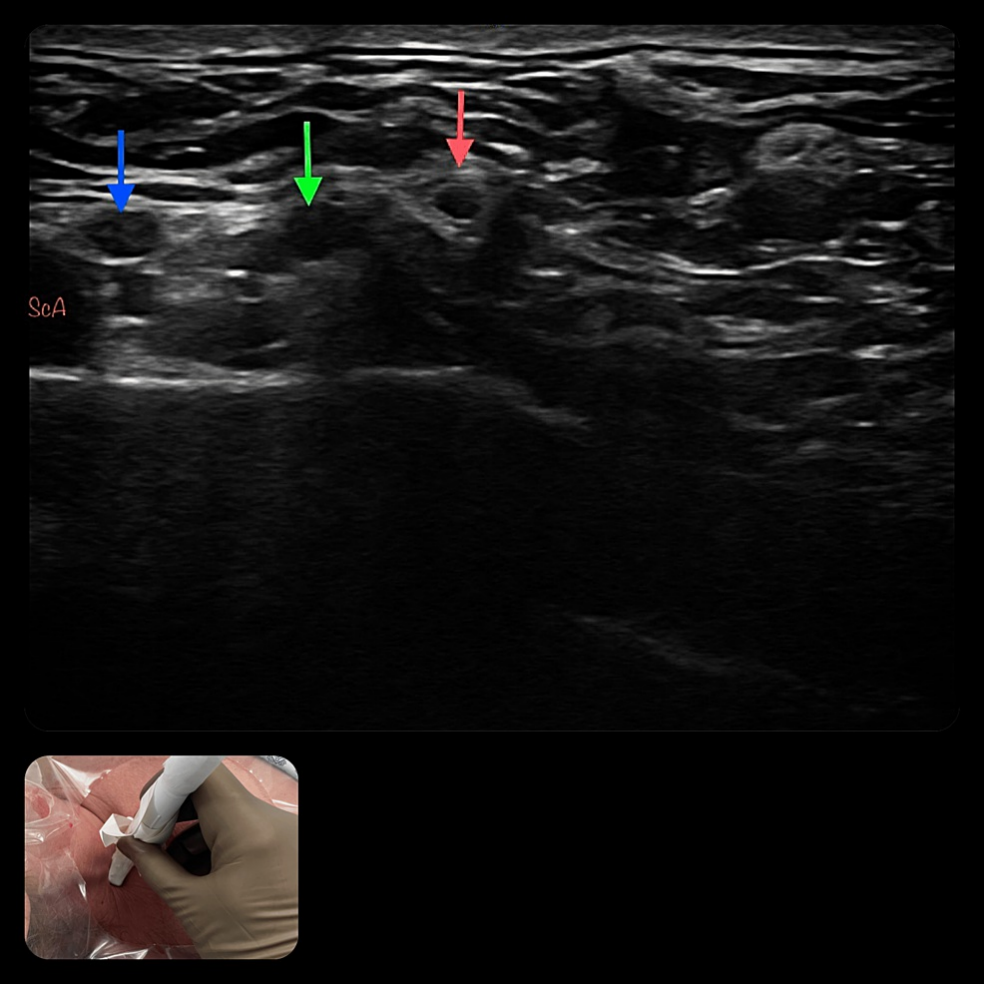
Ultrasound probe position and image for anterior suprascapular nerve block ScA: subclavian artery, Blue arrow: anterior division of upper trunk, Green arrow: posterior division of upper trunk, Red arrow: suprascapular nerve

The suprascapular nerve was blocked in the supraclavicular fossa, using the method previously described by Siegenthaler et al. [13]. The patients were placed in a semi-sitting position with their heads facing the opposite side. After sterile preparation using chlorhexidine and draping a linear array transducer (GE, LOGIQ P9 R3, Seongnam-si, Republic of Korea) with a large bandwidth, a multifrequency linear probe (4-14 MHz) was protected by a sterile sheath. This was attached in the transverse plane for the purpose of visualizing the superior trunk along the short axis. The suprascapular nerve was identified at the point at which it branched off from the superior trunk (Figure 1). It was then traced until it coursed below the inferior belly of the omohyoid muscle. Following skin infiltration with 1 ml of 2% prilocaine, a 22-gauge, 5-cm, fully coated, 30-degree back-cut bevel needle (Ultra360 Stimuplex, B. Braun Medical, Inc., Bethlehem, PA, USA) was inserted in line with the probe in a lateral-to-medial orientation in the direction of the suprascapular nerve. Following negative aspiration for blood, the local anesthetic solution was injected for the purpose of circumferential spreading around the neurovascular bundle.

Failed Block Criteria

Some of the criteria that lead to block failure are: (i) a postoperative 30-minute pain score (NRS) of 4 or above despite the rescue analgesic administration protocol; (ii) a postoperative 60-minute pain score (NRS) of 4 or above despite the rescue analgesic administration protocol; ​​​​​​​(iii) morphine consumption of 20 mg or more as an additional analgesic until the sixth hour postoperatively.

The Function of the Diaphragm Evaluation

Patients were asked to assume a semi-seated position. Briefly, a 3.5-5 MHz curvilinear probe (GE, LOGIQ P9 R3, Seongnam-si, Republic of Korea) was installed beneath the costal margin between the anterior axillary and mid-clavicular lines. A medial, dorsal, and cephalic probe orientation was employed for the purpose of visualizing the posterior one-third of the hemidiaphragm. The liver was employed as an acoustic window for the right side and the spleen for the left side. The ultrasound device was set to motion mode. As a result, the diaphragm appeared in the form of a white hyperechoic line, which undulated as the respiratory cycle progressed. The diaphragmatic excursion was defined as a craniocaudal movement of the diaphragm and was calculated in centimeters during a voluntary "sniff test" prior to the administration of regional anesthesia (basal state). It was then measured once again 30 minutes after block administration. The mean value of three consecutive measurements of the diaphragmatic excursion was recorded at each recording time.

Study Stopping Rules

Consistent with previous non-probability sequential dosing employed in studies reporting similar binary outcomes, we estimated that at least 10 independent negative-positive up-and-down deflections would be needed to calculate the MEAV50 [14,15].

Statistical analysis

Statistical analysis was performed on IBM SPSS Statistics for Windows, Version 26.0 software (IBM Corp., Armonk, NY, USA). Descriptive statistics are summarized as counts and percentages for categorical variables and as mean and standard deviations or standard error and median (range) for others. Ninety-five percent confidence intervals are also given. Differences among more than two groups for ordinal or non-normally distributed continuous variables were evaluated using Kruskal-Wallis variance analysis. When the p-value from the Kruskal-Wallis test statistics was statistically significant, Dunn’s test was used to determine which group differed from the others. A one-way ANOVA was used to evaluate differences among the six groups for normally distributed continuous variables. Duncan’s test was used for multiple comparisons in the case of a significant difference among groups. A repeated-measures ANOVA was used to test differences within and between groups and the interaction between them. The effective volumes of local anesthetic that would be required to produce a complete sensory block in 50% (ED50) and 95% (ED95) of the patients were calculated using probit transformation and logistic regression. p values lower than 0.05 were considered significant.

## Results

Sixty-seven patients were included in the study. The patients’ demographic and operative characteristics are given in Table 1.

**Table 1:** Demographic data and operation characteristics ASA-PS: American Society of Anesthesiologists Physical Status, NRS: Numerical Rating Scale. Data are presented as number of patients (%)/mean values ‪± SD.

Age (years)		54.12 ± 9.22
Gender	Male	58.83
	Female	41.17
Weigh (kg)		75.26 ± 13.24
Height (cm)		171.68 ± 7.79
Body mass index (kg m^−2^)		25.41 ± 3.49
ASA-PS	I	35.29
	II	58.82
	III	5.89
Preoperative baseline pain at rest (NRS)		3.76 ± 2.3
Preoperative baseline pain at activity (NRS)		6.62 ± 2.23
Duration of surgery (min)		91.21 ± 15.3

**Figure 2:**
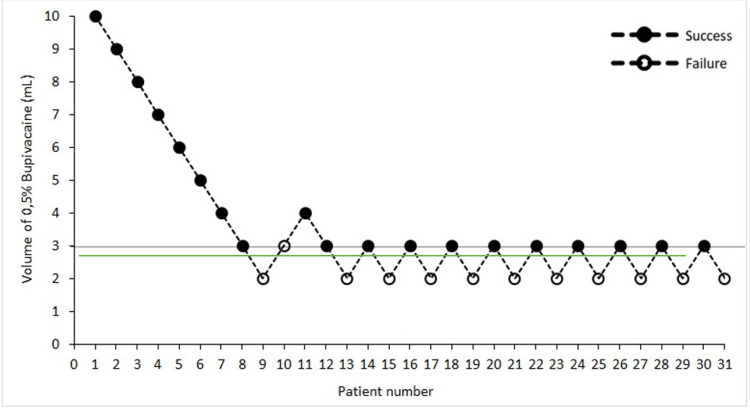
Up‐and-down graph showing the volume of 0.5% bupivacaine given per patient Green line: minimum effective volume (ED50).

The up-and-down methodology of Dixon and Massey was applied with a starting dose of 10 ml of local anesthetic for an anterior suprascapular nerve block. The study was scheduled for termination in the case of 10 consecutive successful and unsuccessful blocks. The results are shown in Figure 2. The ED50 and ED95 calculated with the probit transformation and logistic regression analysis were 2.646 (95% CI, 0.877-2.890) and 3.043 ml (95% CI, 2.771-4.065), respectively. Calculated fit statistics for the probit regression were chi-square (df:7) = 0.067 and a p-value of 1.000.

**Figure 3:**
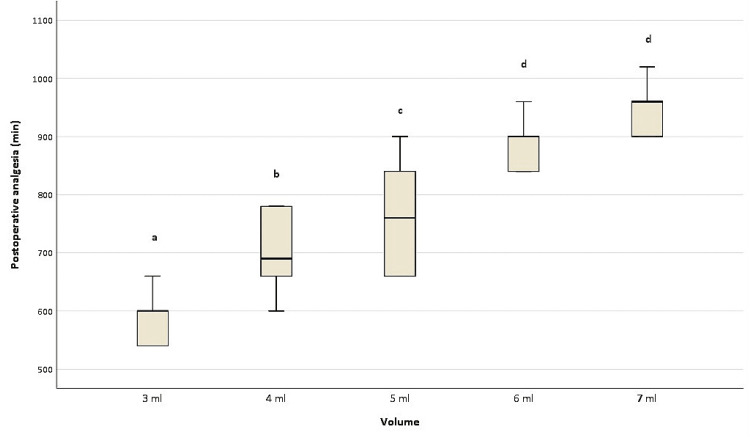
Times to first analgesic requirement pain post-nerve block associated with local anesthetic volumes Means/medians shown with the same letter (such as a and b) are the same (p>0.05), while those with different letters are statistically different (p<0.05) (from one another; no statistically significant difference was observed between 6 ml and 7 ml).

Times to first pain after nerve block related to local anesthetic volumes are shown in Figure 3. The duration of postoperative analgesia decreased in line with the amount of local anesthetic used. No statistical difference was observed between the 6 ml and 7 ml groups. However, statistical significance was observed among other volumes. Postoperative fentanyl in the PACU was used only in nerve block patients who received 2 ml of local anesthetic. The average fentanyl dose was 122.91 µg.

**Figure 4:**
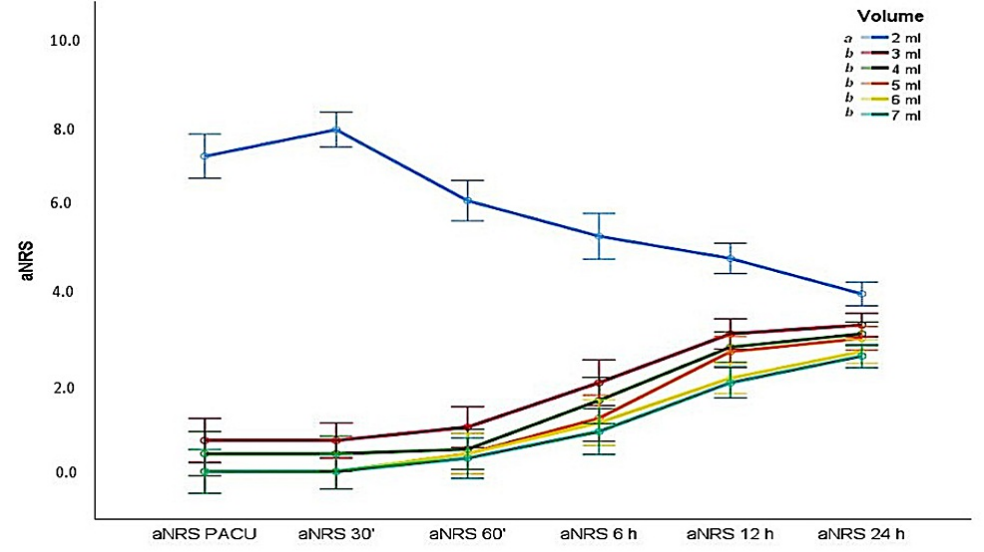
Postoperative 0–24 hour activity pain scores according to local anesthetic volumes (mean values ‪± SD)‬‬‬‬‬‬‬‬‬‬‬‬‬‬‬‬‬‬‬‬‬‬‬‬‬‬‬‬‬‬‬‬‬‬‬‬‬‬‬‬‬‬‬‬‬‬‬‬‬‬‬‬‬‬‬‬‬‬‬‬‬‬‬‬‬‬‬‬‬‬‬‬‬‬‬‬‬‬‬‬‬‬‬‬‬‬‬‬‬‬‬‬‬‬‬‬‬‬‬‬‬‬‬‬‬‬‬‬‬‬‬‬‬‬‬‬‬‬‬‬‬‬‬‬‬‬‬‬‬‬ aNRS: activity pain score. Means/medians shown with the same letter (such as a and b) are the same (p>0.05), while those with different letters are statistically different (p<0.05).

**Figure 5:**
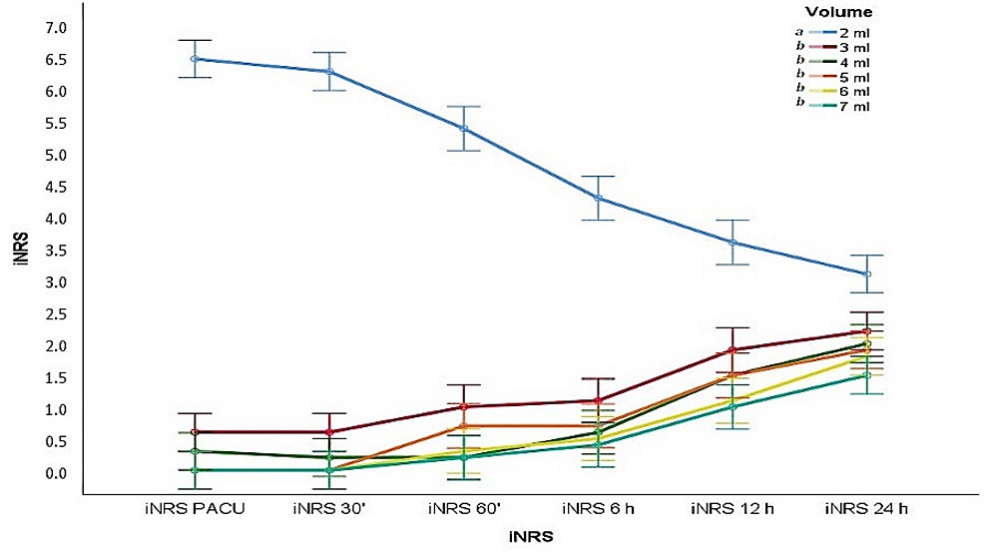
Postoperative 0–24 hour resting pain scores according to local anesthetic volumes (mean values ‪± SD)‬‬‬‬‬‬‬‬‬‬‬‬‬‬‬‬‬‬‬‬‬‬‬‬‬‬‬‬‬‬‬‬‬‬‬‬‬‬‬‬‬‬‬‬‬‬‬‬‬‬‬‬‬‬‬‬‬‬‬‬‬‬‬‬‬‬‬‬‬‬‬‬‬‬‬‬‬‬‬‬‬‬‬‬‬‬‬‬‬‬‬‬‬‬‬‬‬‬‬‬‬‬‬‬‬‬‬‬‬‬‬‬‬‬‬‬‬‬‬‬‬‬‬‬‬‬‬‬‬‬ iNRS: resting pain score. Means/medians shown with the same letter (such as a and b) are the same (p>0.05), while those with different letters are statistically different (p<0.05).

Pain scores during the first postoperative 24 hours are shown in Figures 4-5. Similar results were observed, except for the failed block volume of 2 ml. Although this was not statistically significant, pain scores increased gradually in patients who had been successfully blocked, depending on the block duration.

**Figure 6:**
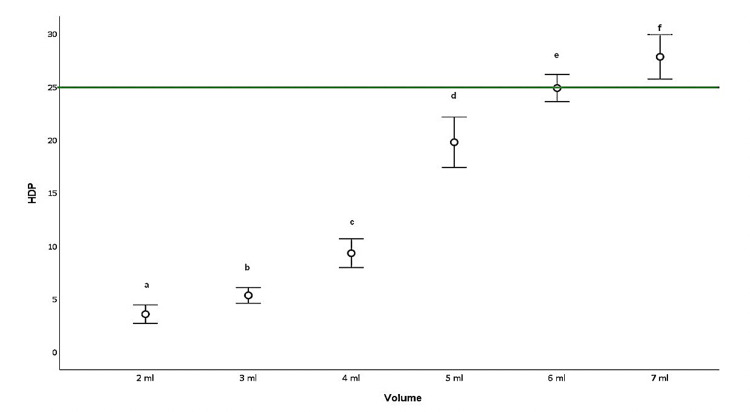
Hemidiaphragmatic paralysis rates according to local anesthetic volumes (mean values %) HDP: hemi-diaphragmatic paralysis. Means/medians shown with the same letter (such as a,b) are the same (p>0.05), while those with different letters are statistically different (p<0.05).

Ten consecutive measurements yielded 10 separate results for patients using 2 ml and for those receiving 3 ml. In order to equalize these numbers, nine additional patients receiving 7, 6, 5, and 4 ml each were also enrolled in the study. Ten patients receiving 7, 6, 5, 4, 3, and 2 ml doses of local anesthetic (a total of 60 patients) were thus finally examined, and their diaphragm functions were evaluated before and 30 minutes post-block. The three consecutive results were then averaged. The observed HDP rates are shown in Figure 6. When complete paralysis was defined as 75% or above, partial paralysis as 25-50%, and no paralysis as 25% or less, volumes of 6 ml or lower appeared to cause no paralysis for the anterior suprascapular nerve block. In particular, volumes of 4 ml or less resulted in HDP rates below 10%. No symptomatic dyspnea developed in any of the patients.

## Discussion

Based on Dixon and Massey's methodology, the minimum effective analgesic volume for anterior suprascapular nerve block in shoulder arthroscopy in the present study was 3 ml. During our investigation of the minimum effective volume for anterior suprascapular nerve block, 10 consecutive successful and unsuccessful postoperative analgesia results were employed, and the patients' diaphragmatic functions were also examined. The results of this examination revealed an HDP rate below 10% in patients administered 3 ml. Higher HDP rates were observed in patients receiving higher volumes, while HDP rates decreased with each reduction in volume. The numbers of patients receiving 7, 6, 5, and 4 ml were therefore raised to 10 each for each volume, and their diaphragm functions were also examined. According to our results, the HDP ratio was below 25% at doses of 6 ml or less for an anterior suprascapular nerve block.

Regional anesthesia techniques play a significant role in eliminating severe postoperative pain and discomfort problems after shoulder surgery. ISBB remains the gold standard method due to its powerful and proven efficacy. However, the use of ISBB in patients with respiratory problems is limited due to HDP and ipsilateral phrenic nerve palsy. Involuntary phrenic nerve block and accompanying respiratory problems may be seen due to the proximity between the phrenic nerve and the brachial plexus. This is especially pronounced in ISBB, and alternative methods have therefore been investigated. The distance between the phrenic nerve and the ventral ramus of C5 varies at different levels of the neck. While the mean distance is 0.20 cm at the cricoid level, the distance increases below the cricoid cartilage. The distance between the phrenic nerve and brachial plexus increases by approximately 3 mm for every centimeter caudal to the cricoid cartilage [16]. The basis of diaphragmatic-sparing nerve blocks, therefore, consists of a blockade of the more distal and caudal branches.

Diaphragm-sparing blocks have been developed, and alternative methods are increasing in popularity. The most popular of these seem to be the anterior suprascapular nerve block (ASSB), supraclavicular block (SCB), upper trunk block, shoulder block, and costoclavicular block (CCB) [17-19]. Various randomized controlled studies have been conducted to reduce local anesthetic volumes in regional anesthesia techniques used in shoulder surgery. Aliste et al. [20] reported an HDP rate of 0% for CCB in their study comparing CCB and ISB. However, this is the only study to report a figure of 0%. Wong et al. [21] conducted a minimum effective volume study for CCB and reported a MEV90 of 20.9 ml for CCB. This reported volume appears to correlate with other studies involving CCB. Mittal et al. [22] investigated the surgical anesthesia dose for ISB and reported a minimum effective volume of 8.64 ml for 0.5% ropivacaine. Studies of diaphragm-sparing nerve block have generally reported higher volumes of local anesthetic used for interscalene block than the reported MEV-ISBB. This can have important consequences for diaphragm protection. The minimum effective volume for the anterior suprascapular nerve block in the present study was 3 ml. In order to achieve consistency with other minimum effective volume studies, we used Dixon's up-and-down methodology. Maikong et al. [23] conducted a cadaveric study on suprascapular nerve block and reported a MEV90 dose of 4.2 mL without phrenic nerve involvement. As those authors stated, cadavers may differ from live patients in terms of facial resistance to methylene blue/local anesthetic diffusion, and there may be differences in dye volume and effectiveness. However, we think that the minimum effective dose in living patients in the present study is close to the value reported in the cadaver study, a finding that confirms the value of cadaver studies.

Ferre et al. [24] compared anterior and posterior suprascapular nerve blocks. Those authors used 10 ml of 0.375% ropivacaine and reported a 5% complete and 31% partial HDP rate for the anterior suprascapular nerve block. The authors also recommended that volume studies be performed for suprascapular nerve block in terms of diaphragm function. Consistent with this result, local anesthetic volumes of 6 ml or less for anterior suprascapular nerve block were capable of preserving diaphragm function in the present study. Anterior suprascapular nerve block has been compared with ISBB in several studies and has been shown to yield an ISBB-equivalent or excellent quality of postoperative analgesia [25-28]. The present study found a very long postoperative analgesia time, even at doses as low as 3 ml.

Renes et al. [29] studied the minimum effective volume for ISBB and pulmonary function at the investigated doses. Those authors used the up-and-down methodology of Dixon and Massey starting at 6 ml and determined a minimum effective dose for ISBB of 2.9 ml. They also examined HDP doses using the up-and-down method employed. The authors observed no deterioration in pulmonary functions in any patients until the second hour after surgery. Similar to the present study, that research investigated the effective dose and diaphragm functions using the up-and-down methodology. They reported significant HDP rates due to catheter use after 24 hours. Significant dose-dependent HDP rates were also determined in the present study.

Limitation

This study has several limitations. First, the up-and-down method of Dixon and Massey is designed to calculate the minimum effective volume (ED50) with a limited number of patients. The calculated ED95 value should therefore be considered only a rough approximation. Second, the suprascapular nerve block was performed under ultrasound guidance only. The nerve stimulator was not used for secondary confirmation. Third, the patients were not evaluated in terms of sensory block. Finally, dose-dependent hemi-diaphragmatic paralysis and postoperative analgesia times were evaluated in a limited number of patients.

## Conclusions

In conclusion, to the best of our knowledge, this is the first minimum effective volume study involving ASSB. An anterior suprascapular nerve block provides effective postoperative analgesia in shoulder arthroscopy. A minimum effective volume (ED50) of 2,646 ml for postoperative analgesia was determined in this study. Based on diaphragm functions, the volume that yielded an HDP rate of 25% and below was 6 ml or less. We, therefore, recommend using a volume of 6 ml or less in order to achieve diaphragm-sparing features for anterior suprascapular nerve blocks. We think it will be important to investigate the minimum effective dose and dose-dependent HDP rates for other diaphragm-sparing nerve blocks in the future.
